# Impact of Altered Breathing Patterns on Interaction of EEG and Heart Rate Variability

**DOI:** 10.1177/0972753120950075

**Published:** 2020-11-09

**Authors:** Meenakshi Sinha, Ramanjan Sinha, Jayshri Ghate, Gaurav Sarnik

**Affiliations:** 1 Department of Physiology, All India Institute of Medical Sciences, Raipur, Chhattisgarh, India; 2 Department of Medicine, JIPMER, Puducherry, India

**Keywords:** Theta wave, HRV, apnea, hyperventilation, slow deep breathing

## Abstract

**Background:**

Altered pattern of respiration has been shown to affect both the cardiac as well as cortical activity, which is the basis of central–autonomic dual interaction concept. On the other hand, effect of this association between altered breathing with slow cortical activity, that is, electroencephalography (EEG) theta waves (associated with learning and relaxed alertness) on the cardiac autonomic balance is largely unclear.

**Objective:**

The study aims to understand this interaction in response to altered respiratory patterns, for example, voluntary apnea, bradypnea, and tachypnea in terms of EEG and heart rate variability (HRV) correlates in normal healthy subjects.

**Methods:**

This study was conducted on 32 adult male subjects. EEG from F3, F4, P3, P4, O1 and O2 cortical areas and Lead II electrocardiography for HRV analysis was continuously recorded during aforesaid respiratory interventions. Power spectral analysis of EEG for theta waves and HRV measures, that is, RMSSD, pNN50, HF, LF, and LF/HF was calculated as % change taking resting value as 100%.

**Results:**

Apnea caused decrease in theta power, whereas an increase in LF/HF was observed in HRV. Bradypnea on the other hand, did not elicit any significant change in power of theta waves. However, decreased RMSSD and pNN50 were observed in HRV. Tachypnea led to increase in theta power with HRV depicting significantly decreased RMSSD and pNN50. Besides, significant correlation between EEG and HRV measures was found during tachypnea, which shifted toward posterior cortical sites as compared to resting condition.

**Conclusion:**

Various altered respiratory patterns caused either depressed parasympathetic or increased sympathetic output, whereas increased theta power along with posterior shift of correlation between theta power and HRV measures observed during post tachypnea might be due to involvement of global brain areas due to respiration-coupled neuronal activity. Thus, a definite link between cortical activity and autonomic output in relation to altered respiratory patterns may be suggested.

## Abbreviations

ANS: autonomic nervous system

CAN: central autonomic network

CNS: central nervous system

EEG: electroencephalography

ECG: electrocardiography

FFT: fast Fourier transformation

HF: high frequency

HRV: heart rate variability

LF: low frequency

pNN50: percent of pairs of adjacent RR interval with a difference of more than 50 msec

RMSSD: square root of mean square differences of consecutive RR intervals

RSA: respiratory sinus arrhythmia

## Introduction

Various neuroimaging studies establishing functional interaction between autonomic nervous system (ANS) and cortical processes involved in consciousness and attention has supported the central autonomic network model for explaining ANS–CNS (central nervous system) interaction.^1 –4^ This interaction forms the basis for the dynamic nature of homeostatic processes against the physiological or pathological challenges to the body.^5^ In this context, heart rate variability (HRV), reflecting the alteration in RR intervals over time, is being used widely for studying the functional link between cardiovascular system and CNS.^6,7^

On lines of the above concept, output of one such autonomically regulated system, that is, respiratory system has been extensively studied to assess the ANS–CNS link. It is quite obvious that altered respiratory pattern due to voluntary control or presence of some disease conditions (e.g., chronic obstructive pulmonary disease (COPD), asthma, or fibrosis, etc.) leads to significant alteration in the autonomic states in terms of sympathetic and parasympathetic responses.^8–10^ Also, hypoxemia, hypercapnia, or hypocapnia induced by the altered respiratory patterns have been shown to elicit several hemodynamic changes, which are also reflected in altered cortical functions.^11,12^ There is widespread presence of respiration-coupled neuronal activity, which reverberate to the whole brain^13^ and has been linked to inter-regional communication.^14^ Besides various other activities, production of slow theta waves at the cortex is responsible for synchronization of neuronal activity.^15^

In this context, recent studies have documented direct link between slow nasal respiration and slow brain rhythms leading to increased delta-theta EEG activity.^11,13^ Such slow deep breathing induced slow EEG activities have been associated with functional state of alert relaxation,^16^ enhanced cognitive processing,^17,18^ and meditative practices.^13^ Deep breathing, besides producing central-largest cortical topography, also leads to increased activation of the parasympathetic nervous system.^17^ Increased correlation has been reported between HRV measures and alpha/theta rhythm of EEG during event related attention exercises.^19^ On the other hand, hyperventilation, which is classically used as an activation method during EEG recording, has been found to provoke physiological slowing of brain rhythms in the range of delta and theta activity.^20^

However, interaction of various signals to regulate the cortical activity remains to be explored. Scant literature is available to correlate the autonomic changes with the slow waves of cortex, mainly theta activity simultaneously during altered respiratory patterns in normal healthy subjects. Therefore, present work aim to assess influence of altered respiratory patterns, that is, voluntary breath-holding (apnea), deep and slow breathing (bradypnea), and hyperventilation (tachypnea) on the slow theta activity (by EEG analysis) with the parallel cardiac autonomic features (in terms of HRV) in the same platform, which might have important role in entraining central autonomic networks in healthy individuals.

## Methods

In this self-controlled prospective study, we recruited 32 healthy adult male volunteers of age group between 18 and 24 years, after obtaining Institutional Ethics Committee clearance. Subsequently, written informed consent followed by detailed medical history was taken from each subject for selection of subjects as per criteria of exclusion and inclusion. Individuals with medical history affecting autonomic function, presence of any psychological or neurological disorder, smoking habit, alcohol consumption/respiratory illnesses/hypertension/diabetes mellitus, etc., were not included.

*Recording of EEG*: International electrode placement (10–20 system) was used to record EEG from frontal, parietal, and occipital regions (F3, P1, O1 as left and F4, P2, O2 as right leads) bilaterally with reference electrodes placed on the left and right earlobes (A1 and A2). The impedance of each electrode was kept at <5 kΩ. Digital EEG machine (Recorder & Medicare System, India) was connected with these electrodes for EEG acquisition. The recorded raw signals were digitized and then put for fast Fourier transformation (FFT) with the help of inbuilt software for power spectral analysis to calculate power of frequency spectrum of EEG waves.

*Recording of ECG for HRV analysis*: Lead II electrocardiography (ECG) was recorded using the standard limb electrode placement. Recording and acquisition of ECG signals was done with the help of inbuilt software followed by short-term HRV analysis for the artifact free record before and after each intervention, using Labchart software (ADInstruments, USA).

*Study design*: All the recordings were done at the laboratory temperature of 26 ± 2 °C in the afternoon, 2–3 hours post prandial. Immediately after arrival to the lab and then after 10–15 minutes, resting blood pressure and heart rate were measured. Then, with instruction to remain completely relaxed, ECG and EEG were simultaneously recorded on the subject with eyes closed in supine position. The recording was done till >50% of alpha activity was observed at the occipital electrode site.^21^ This was followed by the subjects carrying out the simulations of apnea, bradypnea, and tachypnea, as per protocol described later. Following each intervention, at least 15 minutes of resting record were taken so that heart rate and EEG returned to the preintervention condition. Continuous recording of ECG and EEG was done during the whole study period.

### Protocol for Eliciting Altered Breathing Patterns

*Apnea*: Subjects were asked to perform voluntary breath-holding at the end of inspiration phase till he reaches his breaking point as confirmed by the diaphragmatic flutter.^22^

*Bradypnea*: Deep breathing was performed for a period of 3 minutes following a cycle of 6 breaths/minute to produce bradypnea.^23^

*Tachypnea*: Deep and rapid breathing for 3 minutes at a cycle of 30 breaths/minute was performed to produce tachypnea.^8^

*EEG waveform reduction*: Random selection of five artifact free epochs of 6 seconds duration each was done by visually inspecting EEG records during (a) preintervention sessions^21^ and then (b) 0–2 minutes immediately after intervention with an interepoch interval of 20 seconds. With the help of FFT, EEG waveforms were decomposed into their sine wave components in terms of respective frequency bands, that is, alpha (8–12 Hz), beta (15–30 Hz), and theta (4–8 Hz) and absolute power (in uv^2^). However, for the present study, we analyzed the theta power activity only. The recorded powers following each postintervention session for each of the above frequency bands were calculated as percentage (%) change in relation to their respective resting absolute EEG power, so that large variation among the interindividual waves can be addressed. The results have been reported as mean of % change ± standard error (M ± SE).

*Computation of HRV*^24^: As our aim was to look for immediate changes (first 2 minutes) in the EEG and HRV measures as an outcome of altered respiration, we performed short-term HRV analysis of obtained RR intervals. For this, 5 minutes artifact free resting ECG record and 2 minutes postintervention ECG record (as that of EEG record) were selected with the help of software Labchart 6 PRO, ADInstruments, USA, and FFT was used to determine the power spectral density. Thereafter, calculation for time domain indices, that is, pNN50 (percent of pairs of adjacent RR interval with a difference of >50 msec), RMSSD (square root of mean square differences of consecutive RR intervals) and frequency domain indices, that is, LF (low frequency) power, HF (high frequency) power, and total power were done.^4^ As the “normalized unit” (nu) of LF and HF expresses the sympathetic and parasympathetic branches of the ANS in a balanced and regulated manner, we have represented these indices in “nu.”^25^

*Statistical analysis*: Pre- and post-intervention absolute power of alpha, beta, and delta waves at F3, F4, P3, P4, O1, and O2 electrodes were compared by the two-tailed Mann–Whitney *U* test. Correlation between EEG and HRV measures was estimated using Pearson’s correlation test. All statistical analyses were carried out at the significance level ≤.05.

**Table 1.** Correlation Between Theta Power and HRV Indices Across Different Interventions

Theta wave	HRV Indices				
	RMSSD	pNN50	LF	HF	LF/HF
Resting	0.40 (F3)^*^	0.38 (F3) ^*^	NS	NS	NS
Post apnea	NS	NS	NS	NS	NS
Post bradypnea	0.5 (F3) ^*^	NS	NS	NS	NS
Post tachypnea	NS	0.47 (P4)^*^ 0.41(O1)^*^ 0.44 (O2)^*^	–0.46 (O1)^*^ –0.44 (O2)^*^	0.45 (P4)^*^ 0.6 (O1)^***^ 0.54 (O2)^**^	–0.45 (O1)* –0.43 (O2)*

**Source:** Authors.

**Note:** “*r*” represents values for Pearson’s correlation between theta power and HRV indices. Electrode sites depicting left (F3, O1) and right (P4, O2) sides are given in parenthesis. Significance of ‘r’ has been marked as *.

**Abbreviations:** NS, no significant correlation; RMSSD, root mean square of successive differences between adjacent normal RR intervals; pNN50, percentage of number of pairs of adjacent RR interval differing by more than 50 msec; HF, high-frequency component; LF, low-frequency component.

*p < .05; **p < .01; ***p < .001.

## Results

The present study was conducted on 32 normal adult male subjects with a mean BMI of 21.49 ± 2.36. All preintervention resting EEG and HRV values have been expressed as standard units, whereas postintervention data are expressed as % change, resting values being taken as 100%. All postintervention measures of theta power (obtained from EEG) and HRV are calculated in the first 2 minutes of intervention to see the immediate effect of the various respiratory patterns. Part of our EEG findings of alpha and beta waves for these interventions has already been published elsewhere.^26^

**Figure 1.**
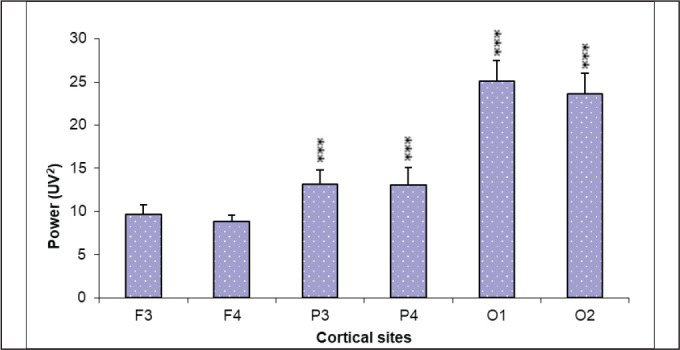
Resting Theta Power

**Legend:** Figure depicts absolute power of theta activity in frontal (F3 and F4), parietal (P3 and P4), and occipital (O1 and O2) leads under resting conditions. Parietal and occipital areas showed significantly increased theta power as compared to their frontal power.

**Source:** Authors.

**Note:** Values are represented as mean ± SE (***p < .001).

**Figure 2.**
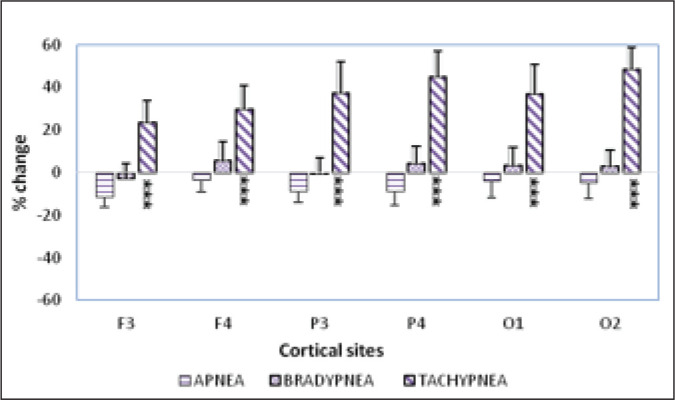
Postintervention Theta Power

**Legend:** Spatial distribution of absolute power of theta activity in terms of % change from resting state following voluntary apnea, bradypnea, and tachypnea. Significantly increased theta activity was seen at all sites as compared to resting state following voluntary tachypnea. However, no significant change was observed after apnea and bradypnea.

**Source:** Authors.

**Note:** Values are represented as mean ± SE (***p < .001).

**Figure 3.**
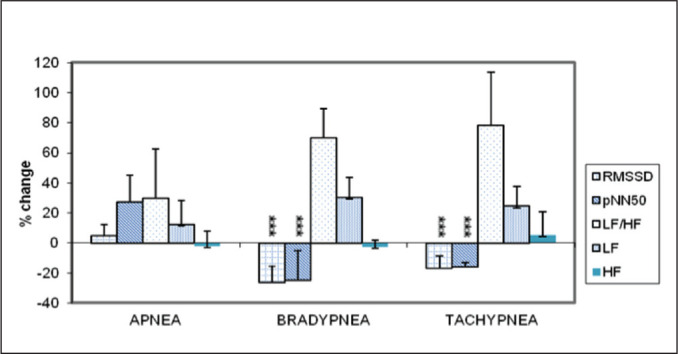
Postintervention HRV Indices

**Legend:** Percentage change in various HRV indices following voluntary apnea, bradypnea, and tachypnea. Significant decrease in RMSSD and pNN50 and marked increase in LF/HF and LF after 3 minutes of bradypnea and tachypnea was seen. Apnea did not cause any significant change.

**Source:** Authors.

**Note:** Values are represented as mean ± SE (***p < .001).

1. *Resting/basal condition*: During resting state, significantly (*p* < .001) higher absolute theta power was recorded at parietal (P3 and P4) and occipital (O1 and O2) areas than frontal site (9.7 ± 4.7 uv^2^ at F3 and 8.87 ± 3.18 uv^2^ at F4) with maximum value at O1 and O2 (25.13 ± 10.6 uv^2^ and 23.66 ± 10.4 uv^2^, respectively) (Figure 1). These cortical activities were found to be bilaterally symmetrical, that is, there was no significant difference between absolute theta power at left (F3, P3, and O1) and right (F4, P4, and O2) sided leads. In terms of HRV, the mean time domain measures, RMSSD and pNN50, in the resting state were 50.89 ± 24.01 msec and 27.04 ± 20.97%, respectively. HF (56.38 ± 15.3 nu) was higher (*p* < .001) than the LF (40.42 ± 17.47 nu) with LF/HF ratio being 1.04 ± 0.32 for the frequency domain indices of HRV. Also, positive correlation (*p* < .05) between theta power and RMSSD and pNN50 was observed only at F3 during the resting state (Table 1).
2. *Post apnea (after voluntary breath-holding)*: Post breath holding/apnea revealed decreased power of theta waves at all the recorded sites (Figure 2), when compared to basal state. However, this decrease was not significant, whereas all the HRV indices (except HF) increased from their resting level within 2 minutes of post apnea, which was maximum for LF/HF, though statistically not significant (Figure 3). No significant correlation was observed between theta waves with any of the time and frequency domain indices (Table 1).
3. *Post bradypnea (after slow deep-breathing)*: Theta power at all the recorded cortical sites did not elicit any marked or significant change following bradypnea (Figure 2). But the time domain measure, that is, RMSSD and pNN50 of HRV showed very significant (*p* < .001) decrease from its resting level after 3 minutes of slow deep breathing, whereas marked increase in LF/HF and LF (though statistically nonsignificant) was observed (Figure 3). The correlation pattern showed significant (*p* < .05) positive correlation between theta activity and RMSSD only at F3 cortical site (Table 1).
4. *Post tachypnea (after hyperventilation)*: Theta activities significantly (*p* < .001) increased at all the frontal, parietal, and occipital electrode sites within 2 minutes of voluntary hyperventilation (Figure 2). Also, a significant decrease in RMSSD and pNN50 values were observed from their resting level, while LF/HF ratio and LF showed marked increase, though statistically nonsignificant (Figure 3). At this time, significant correlation was present between the theta waves and HRV measures (pNN50, HF, LF, and LF/HF) at P4, O1, and O2. pNN50 and HF had a positive correlation, whereas LF and LF/HF always showed negative correlation (Table 1).

## Discussion

The concept of ANS–CNS two-way interaction has paved the path for several studies trying to understand the basis of this link. In this context, many reports have attempted to show how the altered respiratory pattern in different respiratory diseases or during stressful challenging situations influence the cortical activity by linking MRI/EEG findings with cardiac changes and thereby, HRV.^5,17,19^ However, these studies fail to explain adequately the basic link between these two limbs due to the presence of disease/other confounding variables, which are bound to overshadow the normal physiological responses. Therefore, the current report aims to study the physiological responses to altered breathing pattern and also to assess the link between these two systems (cortical and cardiac) in the normal healthy subjects under a common study platform.

*EEG and HRV in resting state*: During resting state, theta power was found to be significantly high posteriorly (Figure 1) with high parasympathetic tone (high HF). In our earlier published report, we have already reported predominant alpha activity mainly posteriorly in the same group of subjects.^26^ These findings are definitely indicative of a true resting state of the subjects as per synchrony of the waves.^21^ Also, the presence of bilateral symmetry of EEG activities is in accordance with the standard protocol of resting EEG recording.^27^

Values of all the indices of frequency domain in our study were found to be similar when compared to Task Force Group Report.^25^ The result indicated a higher parasympathetic/vagal influence (Table 1) because of significantly higher HF (0.15–0.40 Hz) value, which is expected in the resting condition of individuals.^28^ Vagosympathetic modulations are associated with the vagally mediated modulation of heart rate (increasing during inspiration and decreasing during expiration), known as the respiratory sinus arrhythmia (RSA).^29^ However, controversy exist with regard to interpretation of the LF (0.04–0.15 Hz) component, which is now believed to represent both sympathetic and vagal modulation and not only indicate sympathetic tone.^29^

Positive correlation of left frontal (F3) theta waves with time domain measure (RMSSD and pNN50 indicating cardiac parasympathetic control) was evident. It is known that theta activity (4–8 Hz) is seen in the waking adult EEG during relaxed wakefulness maximally in the frontocentral regions, which tends to be somewhat more evident in the midline and temporal derivations.^30^ Therefore, the presence of significant correlation between theta and RMSSD at the left frontal area in our present study corroborates with the fact. High alpha and less beta power were reported by us earlier also in the resting state.^26^ This definitely indicates that increased theta in resting state has relaxing effect (indicated by high parasympathetic activity).

*Post apnea (after breath holding)*: We observed decrease in the theta waves at all the cortical sites starting from frontal to occipital areas following voluntary apnea, which may be explained on the basis that the hypercapnia and hypoxia induced by the apnea may have caused general depressed cortical activity. Marked increase in the LF and LF/HF ratio was observed in our subjects following voluntary apnea, which is an indication of increased sympathetic control over the cardiac system.

Breath holding have been reported to cause activation of both sympathetic and parasympathetic system (i.e., activation of sympathetic limb during holding of breath and parasympathetic activation during late stage of recovery) suggesting a pathophysiological basis of apnea-induced arrhythmias.^31^ Our result is in consonance with these reports thereby suggesting increased sympathetic activity after voluntary apnea.^31,32^

However, no correlation was evident between HRV measures and theta waves following voluntary apnea. This may be due to depressed cortical activity at all cortical areas due to apnea induced hypercapnia and hypoxia.

*Post bradypnea (after slow-deep breathing)*: There was no marked change observed in theta activity following 3 minutes of bradypnea. In our earlier paper,^26^ we had reported decreased alpha activity at the posterior cortical sites in the same recording setup. In this context, a study on pranayamic breathing^33^ simulating the bradypnea state in an individual had shown that stretch induced by voluntary deep breathing generate inhibitory signals, which synchronizes neural control of cardiorespiratory as well as limbic and cortical areas by resetting the autonomic functions.

Also, significantly reduced RMSSD and pNN50 and marked increase in LF and LF/HF signify a decreased parasympathetic and increased sympathetic activity due to 3 minutes of voluntary bradypnea. This is in accordance with the earlier studies^31,32^ showing increased sympathetic activity following controlled breathing pattern. Studies on slow deep breathing techniques have suggested dominance of the parasympathetic tone by increasing HRV and RSA.^34^ Bhastrika pranayama, which is done as a slow rate exercise (respiratory rate 6/min), have been shown to cause strong improvement of autonomic functions by increasing the parasympathetic tone.^35^ However, contradictory reports in terms of increased HF power^17,36,37^ vs no changes^38,39^ or even decreased HF power^40^ are available as an effect of such slow paced pattern of breathing. It could be emphasized in this context that all the reports indicating increased parasympathetic response due to slow controlled breathing were recorded during the slow breathing except the study of Lehrer et al.,^40^ which has reported the response immediately after the session. Therefore, it suggests for altered HRV power in postintervention period, during which respiratory frequency returns to normal. Slow breathing techniques at 9–10 breaths/minute has been seen to cause increase in HF power,^36^ whereas slower breathing at 6 breaths/minute leads to increase in LF power^39–41^ and is usually associated with sympathetic activation.^42^ Our subjects performed 6 breaths/minute to simulate voluntary bradypnea and therefore the findings are confirmed to be in accordance with these literatures.

*Post tachypnea (after mild hyperventilation)*: Voluntary tachypnea led to increased theta power globally and bilaterally (Figure 2). Theta power has been reported to increase whenever individual perform mental tasks/meditation/exposed to external stimuli with decrease in alpha activity.^43^ Such increased theta activity related to phasic event was also observed in our study.

In our present study, the subjects were asked to breath at a rate of 30/minute to simulate tachypnea, which was different from classical hyperventilation^44^ where the subject required to respire as deep and fast as possible like maximum voluntary ventilation, used as an activation technique during EEG recording. We find that the mild hyperventilation in our study simulated states of yoga or meditation and caused relaxation of cortical activity, which was reflected in significant increase in the theta power, more so at posterior cortical sites (Figure 2) with simultaneous increase in frontal alpha power (reported earlier,^26^). Hyperventilation induced physiological slowing of brain rhythms (in delta and theta activity) could be a result of reduced cerebral blood flow due to cerebral hypoxia associated with vasoconstriction in this case.^45^

A significantly decreased RMSSD and pNN50 and marked increased LF/HF as a result of mild hyperventilation are indicative of reduced parasympathetic and increased sympathetic tone. Kox et al.^10^ reported decreased parasympathetic drive to myocardium both during isocapnic and hypercapnic hyperventilation with increased sympathetic activation. Alexopoulos et al.^44^ have also reported exaggerated hemodynamic response due to heightened sympathetic stimulation during hyperventilation. Even kapalbhati (a type of rapid abdominal breathing yogic exercise) has been shown to cause increased LF power and enhanced sympathovagal balance toward sympathetic side with decreased vagal tone that is, HF.^46^ Besides, our study also demanded a high level of concentration while doing HV to maintain the breath rate of 30/minute. Earlier studies have shown that emotional arousal is linked to HRV with decreased HF activity due to increased mental strain, time pressure, and state anxiety,^47,48^ which could be outcome of focusing of attention and associated inhibition of motor activities.^48^ Increased incidences of worrisome events in daily life has been shown to cause reduction in HRV.^49^ Our findings on post-HV HRV changes corroborate these studies.

Theta activities were found to be significantly and positively correlated with measures of parasympathetic (pNN50, HF) and negatively with sympathetic (LF/HF and LF) activity both at parietal and occipital areas bilaterally, post tachypnea. Besides, decreased parasympathetic and increased sympathetic tone correlating with EEG wave patterns were observed mainly at parietal and occipital areas. This was again in contrast to the resting state where correlation existed only at the frontal site, thereby suggesting an altered link between cortical activity and cardiac outflow due to expected changes in pO2, pCO2, and pH caused by hyperventilation induced tachypnea. The reported reverberation of the respiration-coupled neuronal activity to the widespread areas of brain^13–15^ might be the reason for the involvement of posterior cortical areas (parietal and occipital) in the present study, in terms of bringing synchronization between neuronal activity and cardiac output due to hyperventilation. Earlier studies have reported that alpha and theta rhythms of EEG respond differently and in opposite ways with increasing theta power and decreasing alpha power during phasic event related changes.^43^ Another study shows that with increasing task demand, alpha power may desynchronize while theta power synchronize.^50^ In our study also, an increased concentration required on behalf of the subject to maintain the same rate of respiration (30/minute), might be responsible for a stable theta power in parietal and occipital areas.

Therefore, it may be emphasized that our study is corroborating the functional association between brain and cardiac autonomic activity. Higher level cortical structures are known to have reciprocal connections with the subcortical structures, which in turn regulate autonomic input to the heart, leading thereby to its modulation in terms of physiological rhythm of heart rate, that is, HRV. Therefore, HRV has been qualified as independent indicator of CNS–ANS interaction.^50,3^ It may be reiterated here that all our data represent postintervention responses. Therefore, appearance of positive correlation between theta waves with parasympathetic indices and negative correlation with sympathetic indices, which is opposite to that of resting state, may be indicative of body’s response to the interventions toward homeostatic balance, that is, cortical stimulatory interventions leading to compensatory activation of parasympathetic responses and vice versa.

## Conclusion

It is apparent from the discussion that the induced hypoxemia or hypo/hypercapnia due to various altered respiratory patterns (e.g., voluntary apnea, bradypnea, and tachypnea) in normal healthy individuals caused either depressed parasympathetic outflow or increased sympathetic output. Besides, increased theta activity with altered correlation pattern between EEG and HRV measures, which shifted from anterior to the posterior cortex during voluntary hyperventilation, was prominently observed in the present study. Therefore, the impact of altered respiratory pattern induced changes shows definite links between cortical activity and autonomic outflow. The correlation between HRV and EEG findings may also be translated to sensitive cardiac risk markers and also to concurrent hypoxic encephalopathy with predictive potential in related illnesses, for example, COPD, emphysema, etc. However, the present study needs to be conducted with larger sample size including assessment of blood and respiratory gas analysis to conclusively comment on the functional alterations in neural substrates.
